# The evolution of genome size in ants

**DOI:** 10.1186/1471-2148-8-64

**Published:** 2008-02-26

**Authors:** Neil D Tsutsui, Andrew V Suarez, Joseph C Spagna, J Spencer Johnston

**Affiliations:** 1 Department of Environmental Science, Policy and Management, University of California-Berkeley, Berkeley, CA 94720, USA; 2 Department of Animal Biology and Department of Entomology, University of Illinois, Urbana-Champaign, Urbana, IL 61801, USA; 3 Institute for Genomic Biology, University of Illinois, Urbana-Champaign, Urbana, IL 61801, USA; 4 Beckman Institute for Advanced Science and Technology, University of Illinois, Urbana-Champaign, Urbana, IL 61801, USA; 5 Department of Entomology, Texas A&M University, College Station, TX 77843-2475, USA

## Abstract

**Background:**

Despite the economic and ecological importance of ants, genomic tools for this family (Formicidae) remain woefully scarce. Knowledge of genome size, for example, is a useful and necessary prerequisite for the development of many genomic resources, yet it has been reported for only one ant species (*Solenopsis invicta*), and the two published estimates for this species differ by 146.7 Mb (0.15 pg).

**Results:**

Here, we report the genome size for 40 species of ants distributed across 10 of the 20 currently recognized subfamilies, thus making Formicidae the 4^th ^most surveyed insect family and elevating the Hymenoptera to the 5^th ^most surveyed insect order. Our analysis spans much of the ant phylogeny, from the less derived Amblyoponinae and Ponerinae to the more derived Myrmicinae, Formicinae and Dolichoderinae. We include a number of interesting and important taxa, including the invasive Argentine ant (*Linepithema humile*), Neotropical army ants (genera *Eciton *and *Labidus*), trapjaw ants (*Odontomachus*), fungus-growing ants (*Apterostigma*, *Atta *and *Sericomyrmex*), harvester ants (*Messor*, *Pheidole *and *Pogonomyrmex*), carpenter ants (*Camponotus*), a fire ant (*Solenopsis*), and a bulldog ant (*Myrmecia*). Our results show that ants possess small genomes relative to most other insects, yet genome size varies three-fold across this insect family. Moreover, our data suggest that two whole-genome duplications may have occurred in the ancestors of the modern *Ectatomma *and *Apterostigma*. Although some previous studies of other taxa have revealed a relationship between genome size and body size, our phylogenetically-controlled analysis of this correlation did not reveal a significant relationship.

**Conclusion:**

This is the first analysis of genome size in ants (Formicidae) and the first across multiple species of social insects. We show that genome size is a variable trait that can evolve gradually over long time spans, as well as rapidly, through processes that may include occasional whole-genome duplication. The small genome sizes of ants, combined with their ecological, evolutionary and agricultural importance, suggest that some of these species may be good candidates for future whole-genome sequencing projects.

## Background

Genome size is one of the most fundamental genetic properties of living organisms. Moreover, the size of an organism's genome has important practical implications for applications ranging from PCR-based microsatellite genotyping to whole-genome sequencing [1-3].

The genome sizes of invertebrates (particularly insects) remain understudied relative to their abundance and diversity. This is particularly true in light of their impacts on human health, industry, agriculture and science. Of the nearly 1,000,000 described species of insects, genome sizes have been estimated for approximately 453 (0.045%). For comparison, genome size estimates exist for 8.05% of mammals (443 of ca. 5500 species) and 2.06% of birds (206 of ca. 10000 species) [4].

Ants (family: Formicidae) are among the most familiar, abundant and ecologically important of the arthropods [5,6], yet few genomic tools exist for this family of insects, and virtually nothing is known of their genome sizes. Of the nearly 20,000 species of ants that likely exist, the genome size of only one (*Solenopsis invicta*) has been reported [7,8], and the two estimates for this species differ by >146 Mb.

Numerous studies have documented naturally occurring variation in genome size at various taxonomic levels. Clearly, extremely distantly related organisms usually possess genomes of different sizes, as the accumulated effects of genomic expansions and contractions have had ample time to produce measurable differences in genome size. A number of studies have documented genome size variation over more recent evolutionary time-spans – among congeneric species [9], and even among individuals within single species (reviewed in [10,11]).

Ultimately, this genome size variation is determined by the net effects of genome expansion and genomic deletion (reviewed in [10]). Processes that act at the chromosomal scale, such as polyploidy, aneuploidy, and whole genome duplication, can produce rapid and extreme changes in genome size. At smaller scales, the expansion of genomes can be driven by mechanisms such as the proliferation of transposable elements [12-14] or various types of non-coding DNA (reviewed in [15]). For example, both the length [16] and frequency [17-19] of microsatellites (also known as simple sequence repeats {SSRs} or variable numbers of tandem repeats {VNTRs}) are positively correlated with genome size. This is true for taxa as disparate as *Arabidopsis thaliana*, *Caenorhabditis elegans*, *Saccharomyces cerevisiae*, *Drosophila melanogaster*, and *Homo sapiens *(reviewed in [15]). The duplication of coding sequences also appears to play a role in the evolution of genome size, and may be more important for generating phenotypic variation than is widely appreciated [20]. The mechanisms involved in genomic shrinkage, however, remain unclear, and various hypotheses have been proposed to explain this process (reviewed in [21,22]), including reduction of long tandem repeats (LTRs), illegitimate and unequal recombination, and the accumulation of small deletions [23-26]. Overall, the rate of genome size evolution appears to be proportional to genome size, as larger genomes are more likely to experience large or rapid expansions and contractions [27].

The phenotypic correlates and consequences of genome size variation remain murky, but correlative studies in a variety of taxonomic groups have identified a handful of characteristics that co-vary with genome size (reviewed in [10]). For example, a positive relationship between body size and genome size has been reported for several taxonomic groups, including turbellarian flatworms [28], copepods [28], aphids [29] and mosquitoes [30]. Moreover, animals with high metabolic rates, such as flying birds and bats, tend to have smaller genomes, suggesting that metabolism may be a constraint [10,31,32]. Although the ancestors of extant birds possessed genomes that were small.(prior to the evolution of flight, [33]), modern flightless birds, which have presumably been released from flight-associated metabolic constraints, possess genomes that are larger than flying birds [34]. Similarly, flying insects have extremely high mass-corrected metabolic rates [35], and generally have small genomes (reviewed in [10]). At a broad taxonomic level, genome size also appears to be related to developmental life history. Gregory [10,36] proposed, based on data from 18 insect orders, that holometabolous groups (characterized by complete metamorphosis) have smaller genomes than those that are hemimetabolous or ametabolous. Specifically, the holometabolous orders possess genomes smaller than 1C = 2 pg whereas the ametabolous and hemimetabolous taxa, with exceptions [37], possess genomes that span a range from <1 pg to <17 pg. Interestingly, a similar pattern occurs in amphibians – species with rapid metamorphosis typically possess smaller genomes than those characterized by direct development, slower metamorphosis, or neoteny (no metamorphosis) (reviewed in [36]). The adaptive significance of these correlations, if any, remains unclear. Finally, cell size appears to be positively correlated with genome size in a variety of taxonomic groups [10,38].

In this study, we use flow cytometry to estimate the genome sizes for 40 species of ants, collected from 10 of the 20 recognized subfamilies. These data were collected from 173 separate genome size estimations, from 164 individual ants. We also test the hypothesis that genome size is positively correlated with body size, as has been reported in other taxa. Using data from the recent studies of ant phylogeny, we place these results in a phylogenetic context.

## Results and Discussion

**Figure 1:**
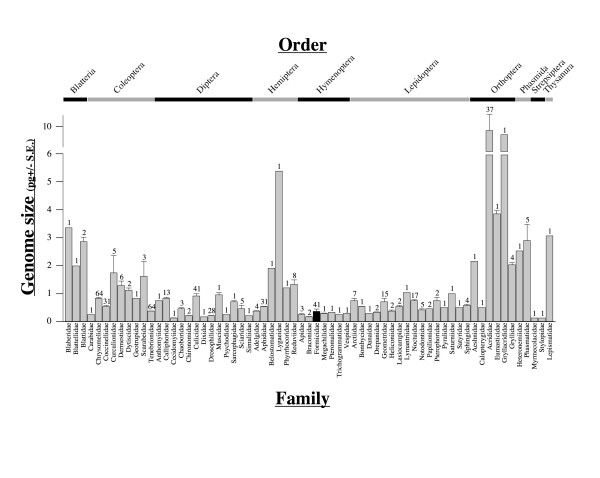
Insect genome sizes (in pg, ± SE), based on data from the Animal Genome Size Database [4]. Ants (Formicidae) are represented by the filled black bar. Each family is labeled at the bottom; the respective orders are shown at the top. Numbers above each bar indicate the number of species in each family for which genome sizes have been estimated. When values from the Animal Genome Size Database were presented as a range, we used the midpoint as the value for that species.

Our data increase the number of published genome size estimates for ants from 1 species (*Solenopsis invicta*) to 41 species. Only three other insect families (Chrysomelidae, Tenebrionidae and Culicidae) have had genomes sizes estimated for more species [4] (Fig. 1). Similarly, only four other orders of insects (Coleoptera, Diptera, Orthoptera and Lepidoptera) have genome sizes estimated for more species [4] (Fig. 1).

Overall, the mean genome size of the ant species examined here was 361.8 Mb (0.37 pg), and values for individual species ranged three-fold, from 210.7 Mb (0.22 pg) in *Cerapachys edentata *to 690.4 Mb (0.71 pg) in *Ectatomma tuberculatum *(Table 1). The subfamilies within which these two species occur (Cerapachyinae and Ectatomminae, respectively) also had the most extreme genome sizes of the ant subfamilies, but we only estimated genome sizes for a single species within each of these subfamilies. For subfamilies represented by more than one exemplar, the Dolichoderinae had the smallest mean genome size (289.1 Mb; 0.30 pg) and the Ponerinae possessed the largest (489.1 Mb; 0.50 pg). The small size of ant genomes appears to be similar to non-ant Hymenoptera. For example, the wasp, *Polistes dominulus*, possesses a genome size of 301.4 Mb (0.31 pg) [8], and the genome size of the honeybee, *Apis mellifera*, is 262 Mb (0.27 pg) [39].

**Table 1:** Sample collection information and estimated genome sizes for 40 species of ants, arranged by subfamily.

Subfamily	Species	1C Genome Size (Mb)	SE	#	Collection Info
Amblyoponinae					
	*Amblyopone pallipes*	335.5	1.2	3	USA: Indiana
	**MEAN**	335.5			
Ponerinae					
	*Dinoponera australis*	554.7	1.7	3 (1) ^1^	ARGENTINA: Misiones Prov.
	*Odontomachus brunneus*	429.8	1.7	5	USA: Florida
	*Odontomachus bauri*	477.3	0.7	5 (3) ^1^	COSTA RICA: La Selva
	*Odontomachus clarus*	414.0	2.0	3	USA: Arizona
	*Odontomachus chelifer*	523.2	11.7	4 (2) ^1^	ARGENTINA: Misiones Prov.
	*Odontomachus haematodus*	496.5	7.0	5	ARGENTINA: Corrientes Prov.
	*Odontomachus cephalotes*	425.0	13.0	4	AUSTRALIA: Queensland
	*Ponera pennsylvanica*	591.9	1.3	2	USA: Illinois
	**MEAN**	489.1			
Myrmicinae					
	*Atta cephalotes*	300.1	1.1	2	PANAMA: Gamboa
	*Atta columbica*	298.8	0.8	2	PANAMA: Gamboa
	*Apterostigma dentigerum*	636.4	7.1	7	COSTA RICA: La Selva
	*Crematogaster hespera*	275.9	1.8	3	USA: California
	*Eurhopalothrix procera*	377.2	2.1	4	AUSTRALIA: Queensland
	*Messor andrei*	253.5	1.4	8	USA: California
	*Myrmecina americana *A	250.7	0.4	2	USA: Illinois
	*Myrmecina americana *B	302.9	1.4	8	USA: Illinois
	*Pheidole hyatti*	326.7	11.5	3	USA: California
	*Pogonomyrmex badius*	262.8	11.9	4	USA: Florida
	*Pogonomyrmex californicus*	249.5	0.8	4	USA: California
	*Pogonomyrmex coarctatus*	282.9	2.5	6	ARGENTINA: Santa Fe Prov.
	*Pyramica rostrata*	278.6	1.0	5 (4) ^1^	USA: Illinois
	*Sericomyrmex amabilis*	440.7	2.1	7	COSTA RICA: La Selva
	*Solenopsis xyloni*	472.3	1.3	3	USA: California
	*Tetramorium caespitum*	256.4	1.0	6	USA: Illinois
	**MEAN**	329.1			
Formicinae					
	*Camponotus pennsylvanicus*	322.8	4.6	5	USA: Illinois
	*Camponotus castaneus*	304.2	2.1	5	USA: Illinois
	*Formica pallidifulva*	385.1	8.7	6	USA: Illinois
	*Lasius alienus*	307.7	1.8	5	USA: Illinois
	*Prenolepis imparis*	296.2	2.2	4	USA: California
	**MEAN**	323.2			
Dolichoderinae					
	*Dorymyrmex bicolor*	249.0	-	1	USA: California
	*Linepithema humile*	250.8	1.5	8	USA: California
	*Liometopum occidentale*	282.0	1.0	3	USA: California
	*Tapinoma sessile*	374.4	1.5	4	USA: California
	*Tapinoma sessile*	593.1	-	1	USA: California
	**MEAN **^2^	289.1			
Pseudomyrmicinae					
	*Pseudomyrmex gracilis*	387.0	1.5	2	USA: Florida
Ectatomminae					
	*Ectatomma tuberculatum*	690.4	7.0	3 (1) ^1^	COSTA RICA: La Selva
Ecitoninae					
	*Eciton burchelli*	263.9	2.1	4	COSTA RICA: La Selva
	*Labidus coecus*	365.8	8.6	4	COSTA RICA: La Selva
	**MEAN**	314.9			
Myrmeciinae					
	*Myrmecia varians *gp.	269.5	12.0	2	AUSTRALIA: Queensland
Cerapachyinae					
	*Cerapachys edentata *gp.	210.7	1.5	8	AUSTRALIA: Queensland

^1 ^When replicates were taken from the same individual, the total number of individuals used is shown in parentheses

^2 ^Excludes *Tapinoma sessile *individual with 593.1 MB genome.

Ants, in general, appear to possess small genomes relative to other insect families (Fig. 1). Of the 60 families for which sufficient data exist, only 16 possess a mean genome size that is smaller than that of the Formicidae. Moreover, 10 of these 16 families are represented by a single species, and 3 have estimates from only two species.

Our estimate of genome size for the fire ant, *Solenopsis xyloni*, was 472.3 Mb (0.48 pg). This appears to be substantially smaller than the genome size of the congeneric red imported fire ant, *S. invicta*, estimated at 753.3 Mb using flow cytometry [8].

For one species, *Myrmecina americana*, we found individual workers with two distinctly different genome sizes living within the same colony. Nine of the individuals sampled possessed a genome size that was near 300 Mb, but two individuals possessed genomes of about 250.7 Mb. This could represent true intraspecific variation in genome size, or may indicate the presence of an undescribed cryptic social parasite, which has been reported for this species (Stefan Cover, pers. comm.). In our analyses, we treat these classes of individuals as two different samples.

Similarly, the two *Odontomachus chelifer *individuals analyzed possessed genomes that differed by 39.9 Mb. This difference is unlikely to be measurement error, as we repeated our measurements for both of these individuals and found similar values. For this species, we included both values in our analyses, since we had no *a priori *reason to expect the presence of two different species.

The largest proportion of the total variance (58%) was observed among subfamilies, followed by 36% of the total variance apportioned among genera (Table 2). Only 3.8% of the total variance was apportioned among species, but this is likely a consequence of the relatively small number of species sampled within some of the subfamilies and genera.

**Table 2:** Variance components.

Taxonomic level		% variance
Among subfamilies	18,726	58.0
Among genera	11,613	36.0
Among species	1,222	3.8
Error	727	2.2

The TFSI test on the genome size values of the terminal taxa yielded P-values of 0.20, whether or not outliers were excluded. We then used independent contrast values to test the relationship between body size and genome size. Independent contrast analysis of genome size vs. body size showed no significant correlation between genome size and head width when phylogeny was taken into account (r^2 ^< 0.002, P = 0.82, P = 0.84 with outliers excluded). Re-running the TFSI test using the contrast data indicated that the use of independent contrasts had successfully reduced any influence of phylogenetic autocorrelation on the data (P > 0.5). Compared to previously studied taxa [28-30], the ants studied here possessed a substantially narrower range of genome sizes, which may have made it more difficult to detect a relationship between genome size and body size.

**Figure 2:**
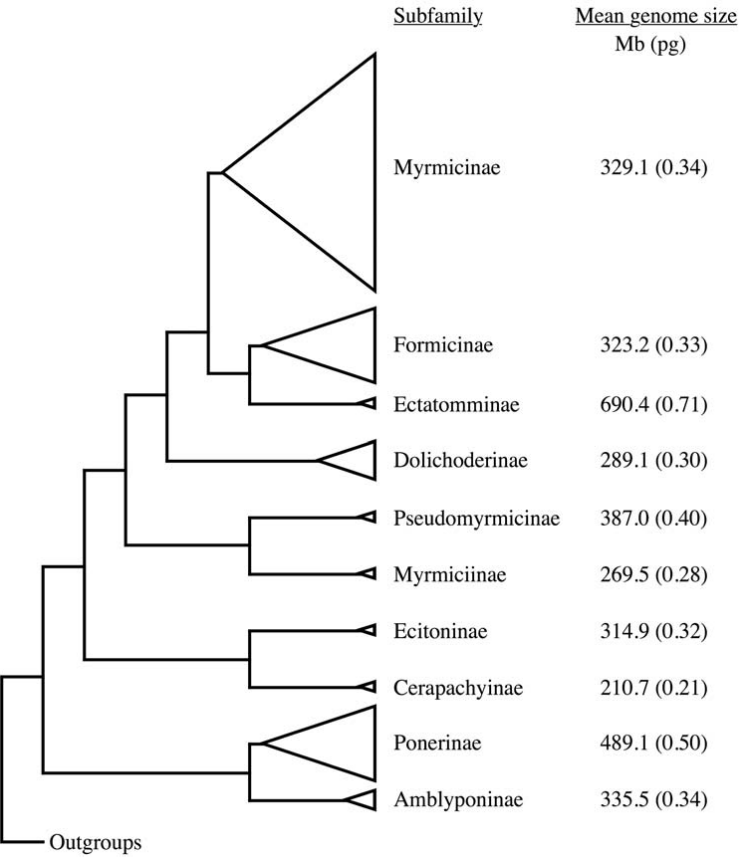
Genome sizes of ant subfamilies. Phylogenetic tree redrawn from Moreau *et al*. (2006) and Brady *et al*. (2006), omitting subfamilies that were not included in this study. The size of each triangle is drawn proportional to the number of species in the respective subfamily.

Comparison of genome sizes among subfamilies suggests that genomes of ants have both expanded and contracted since the origin of ants, approximately 140 million years ago [40,41]. *Amblyopone pallipes*, in the less derived subfamily Amblyoponinae, possesses a genome size of 335.5 Mb (0.34 pg), which is slightly larger than the mean size of the more derived Formicidae, Dolichoderinae and Myrmicinae (Fig. 2). The Ponerinae, on the other hand, possess genomes that are larger than that of *A. pallipes*, suggesting a genomic expansion in this lineage.

Interestingly, we found evidence for genomic expansion, perhaps via whole-genome duplication, in two ant lineages. *Ectatomma tuberculatum*, the sole species sampled from the subfamily Ectatomminae, possesses a genome size of 690.4 Mb (0.71 pg), which is about twice that of the most closely related subfamilies, Formicinae (323.2 Mb; 0.33 pg), Myrmicinae (329.1 Mb; 0.34 pg) and Dolichoderinae (289.1 Mb; 0.30 pg) (Fig. 2). This suggests that a genome duplication may have occurred in an ancestor of *Ectatomma*, potentially as long as 80–90 million years ago [40,41]. More thorough sampling of species in this and closely related subfamilies (such as the sister subfamily, Heteroponerinae) may illuminate more precisely when this large genomic expansion occurred.

Similarly, the genome size of the fungus-growing ant, *Apterostigma dentigerum *(636.4 Mb; 0.65 pg), is about twice that of the most closely related species. *Atta cephalotes *(300.1 Mb; 0.31 pg), *A. columbica *(298.8 Mb; 0.31 pg) and *Sericomyrmex amabilis *(440.7 Mb; 0.45 pg), also fungus-growing ants in the tribe Attini, possess genomes that are about 47%, 47% and 69%, respectively, the size of the *Apterostigma *genome. Because the fungus-growing attines diverged from other ants about 50 million years ago [42] the genomic expansion in *Apterostigma *may have occurred more recently than the one in *Ectatomma*. The history of this genomic expansion, and whether it arose via a whole-genome duplication, could be clarified by a closer examination of these genomes for signatures of genome duplication as well as knowledge of genome size in more closely related species, such as in the genera *Mycocepurus *and *Cyphomyrmex *[43,44].

Although few other studies have examined genome size at the levels of individual, species, genus, and subfamily, recent analyses of spiders and species of Lepidoptera have revealed patterns similar to what we report for the ants. Specifically, within Lepidoptera, genome size varies little within subfamilies, and more substantially among subfamilies and families [45]. Similarly, in spiders most of the variation in genome size occurs within families, and less is partitioned among genera and species [46].

Previous studies have suggested that animals with high metabolic rates, including flying insects, possess relatively small genomes [10]. Although most individual ants are wingless workers, and do not ever fly, ants still have extremely small genomes. This may be because males and queens, in many species, engage in nuptial flights. This pattern may hold for non-flying lineages because they are descended from ancestors who flew [6], and thus had high metabolic rates.

A growing body of research suggests that species that undergo complete metamorphosis possess genomes that are smaller than 2 pg [10,36]. Our data match this pattern: All ants undergo complete metamorphosis and the genome sizes of the species that we examined all fall at the small end of the spectrum. In fact, compared to other insect families, ants (like other Hymenoptera) appear to possess some of the smallest genomes (Fig. 1).

Clearly, within this family, as well as within other taxonomic groups, there are interesting and unexplained patterns of genome size variation. Within the ants, exploration of intergeneric genome size variation within the Attini or inter-subfamilial variation centered on Ectatomminae may illuminate processes involved in the expansion and contraction of genomes across divergent evolutionary time scales. These data may be particularly useful when placed into phylogenetic context using the recent in-depth studies of Moreau *et al*. [40] and Brady *et al*. [41]. Similarly, closer examination of genetic characteristics such as number of transposable elements, intron size, and microsatellite size and number, may elucidate the mechanisms by which genomes expand and contract through time.

Finally, the recent development of genomic tools for ants [47] and whole-genome sequences for other Hymenoptera [39] suggest that similar resources may be on the horizon for other ant species. Because genome size is an important consideration for whole-genome sequencing programs, our genome size data will be useful for guiding selection of candidate ant taxa. Not only will knowledge of genome size be useful in this arena, for the development of ant genomics, but the resulting genomic tools will also inform studies of genome size evolution.

## Conclusion

The total amount of genetic material possessed by an organism is a fundamental feature of its biology. However, we currently know little about the processes that underlie variation in genome size, or even how much variation occurs within and among most taxa. Our study is the first to explore the variation and evolution of genome size in ants, and one of the first (in any system) to examine genome size variation across a range of taxonomic levels, from individuals up to subfamilies. We show that ants, in general, have remarkably small genomes, and that most variation in genome size occurs among subfamilies. Given the ecological, agricultural and economic importance of ants, these findings indicate that many species may be amenable to the development of genomic tools, or even whole-genome sequencing projects. Moreover, the presence of both large- and small-scale variation in genome size (and a well-studied phylogeny) suggests that ants may be useful model systems for exploring the general processes underlying the evolution of genomes.

## Methods

We collected ants from colonies in the field that were discovered by visual searching. When possible, we used multiple individuals from the same colony as replicates for genome size estimates. In some cases, when we were unable to replicate across individuals, we replicated across tissues from the same individual. Genome sizes are presented here as either megabases (Mb) or C-value, which is the haploid nuclear DNA content expressed in picograms (pg) (1 pg = 978 million bases).

To estimate genome sizes, we dissected brains from the ant species under consideration and from the yellow-white strain of *Drosophila melanogaster*.(described in [48]). These were ground together in Galbraith buffer using 15 strokes of the "A" pestle in a Kontes 2 ml Dounce. The mixture was passed through a 50 micron filter, stained with 50 parts per million (ppm) of propidium iodide and run (after 30 minutes in the cold and dark) in a Partek flow cytometer with the laser emitting an exciting light at 514 nanometers (nm). Red flourescence from propidium iodide (intercalated into the DNA of the 2C and 4C nuclei of *Drosophila *and ant) was detected using a high bandpass filter (615 nm). The amount of DNA in the ant was calculated as the ratio of mean channel number of the 2C ant/mean channel of 2C *Drosophila *times 175 Mb. The latter is the genome size of the sequenced strain of *Drosophila *anchored against the fully sequenced *Caenorhabditis elegans *[48]. Overall, we performed 173 genome size estimations from 164 individual ants.

To compare the genome sizes of ants (family: Formicidae) to the genome sizes of other insect families, we compiled data from the Animal Genome Size Database [4]. These data were downloaded from the database in September 2007, when there were 535 estimates of genome sizes from 453 species of insects. Since there were two different estimates for the single ant species represented (*Solenopsis invicta*), we used the value of 753.3 Mb ± 1.8 S.E. (0.77 pg), which was estimated using flow cytometry [8], rather than the smaller value, which was estimated via disassociation kinetics [7]. We used ANOVA, implemented in ProcGLM (SAS), to examine variation in genome size at different taxonomic levels.

One individual of one species, *Tapinoma sessile*, appeared to be a polyploid, and was thus excluded from further analyses (but is shown in Table 1). Four other ants from four different species appeared to be outliers, with genome sizes that were 34.0 – 47.7 Mb larger (in three cases) or smaller (in one case) than the mean genome size for the other representatives of that species. In these cases, we conducted our analyses both including and excluding the outliers.

To test for phylogenetic autocorrelation and to correct for phylogenetically-based non-independence of genome-size data, we used the multi-step approach of Abouheif [49]. First, we used a test of serial independence (TFSI, as implemented in the PI software package, [50]) to determine whether phylogenetic autocorrelation was statistically relevant in our dataset. With that test indicating the possibility of phylogenetic effects on genome size, we used the independent contrasts method of Felsenstein [51] as implemented in the software package CAIC [52]. Taxa were arranged by phylogenetic relatedness of their respective genera according to the phylogenies of Brady *et al*. [41] and Moreau *et al*. [40], which were complementary and congruent with respect to the taxa sampled. Branch lengths were treated as equal due to the combination of data from independently derived phylogenies, and monophyly of genera was assumed for the purposes of determining the branching pattern, as the published phylogenies used different species exemplars than this study in many cases. Head width was used as a proxy for size, and treated as the independent variable, and genome size as the dependent variable for the independent contrast analysis. For genera with multiple species exemplars (e.g. *Odontomachus*), the arithmetic mean of the genome size across all species was used as the value for the genus. Contrast values (30) for genome size were regressed against those for head width. Contrast values were subsequently reanalyzed using the PI software to determine whether the contrast method had successfully produced statistically independent results. All PI and IC analyses were also run using a data set which excluded the outliers described above.

## Abbreviations

Pg = picograms, Mb = megabases, or million base pairs, PCR = polymerase chain reaction, IC = independent contrasts.

## Authors' contributions

NDT and AVS conceived and designed this study and performed the field-work. JSJ performed the flow cytometry to estimate genome sizes. AVS collected the body size data and JCS performed the tests for phylogenetic autocorrelation and independent contrasts. Other analyses were performed by NDT and JSJ. NDT, JCS and AVS provided funding for the research and NDT wrote the manuscript with input from all authors. All authors read and approved the final manuscript.
